# Semi-supervised Learning for Generalizable Intracranial Hemorrhage Detection and Segmentation

**DOI:** 10.1148/ryai.230077

**Published:** 2024-03-06

**Authors:** Emily Lin, Esther L. Yuh

**Affiliations:** From the Department of Radiology & Biomedical Imaging, University of California San Francisco, 185 Berry St, San Francisco CA 94107.

**Keywords:** Semi-supervised Learning, Traumatic Brain Injury, CT, Machine Learning

## Abstract

**Purpose:**

To develop and evaluate a semi-supervised learning model for intracranial hemorrhage detection and segmentation on an out-of-distribution head CT evaluation set.

**Materials and Methods:**

This retrospective study used semi-supervised learning to bootstrap performance. An initial “teacher” deep learning model was trained on 457 pixel-labeled head CT scans collected from one U.S. institution from 2010 to 2017 and used to generate pseudo labels on a separate unlabeled corpus of 25 000 examinations from the Radiological Society of North America and American Society of Neuroradiology. A second “student” model was trained on this combined pixel- and pseudo-labeled dataset. Hyperparameter tuning was performed on a validation set of 93 scans. Testing for both classification (*n* = 481 examinations) and segmentation (*n* = 23 examinations, or 529 images) was performed on CQ500, a dataset of 481 scans performed in India, to evaluate out-of-distribution generalizability. The semi-supervised model was compared with a baseline model trained on only labeled data using area under the receiver operating characteristic curve, Dice similarity coefficient, and average precision metrics.

**Results:**

The semi-supervised model achieved a statistically significant higher examination area under the receiver operating characteristic curve on CQ500 compared with the baseline (0.939 [95% CI: 0.938, 0.940] vs 0.907 [95% CI: 0.906, 0.908]; *P* = .009). It also achieved a higher Dice similarity coefficient (0.829 [95% CI: 0.825, 0.833] vs 0.809 [95% CI: 0.803, 0.812]; *P* = .012) and pixel average precision (0.848 [95% CI: 0.843, 0.853]) vs 0.828 [95% CI: 0.817, 0.828]) compared with the baseline.

**Conclusion:**

The addition of unlabeled data in a semi-supervised learning framework demonstrates stronger generalizability potential for intracranial hemorrhage detection and segmentation compared with a supervised baseline.

**Keywords:** Semi-supervised Learning, Traumatic Brain Injury, CT, Machine Learning

*Supplemental material is available for this article.*

Published under a CC BY 4.0 license.

See also the commentary by Swinburne in this issue.

## Graphical abstract

**Figure:**
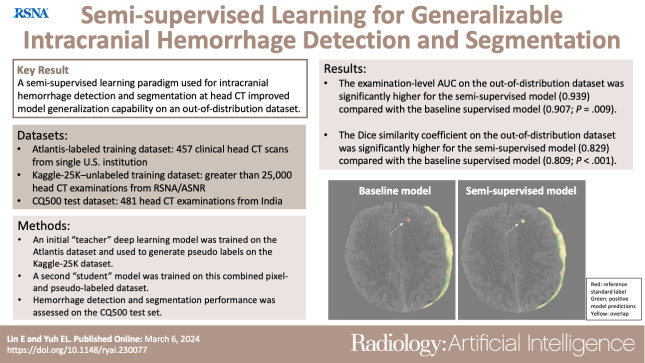

**Summary**

A semi-supervised learning paradigm used for intracranial hemorrhage detection and segmentation on head CT images significantly improved model generalization capability on an out-of-distribution dataset.

**Key Points**

■ The examination-level area under the receiver operating characteristic curve on an out-of-distribution dataset was significantly higher for the semi-supervised model (0.939) compared with the baseline supervised model (0.907) (*P* = .009).
■ The Dice similarity coefficient on an out-of-distribution dataset was significantly higher for the semi-supervised model (0.829) compared with the baseline supervised model (0.809) (*P* = .012).

## Introduction

CT is the brain imaging modality most commonly used to diagnose acute traumatic brain injury. However, CT images can be challenging to interpret, as tiny abnormalities occupying only ~100 pixels in a low-contrast volume of less than 106 pixels must be detected. As CT images are characterized by image artifacts and low signal-to-noise ratios, they are read by experts after years of training. Because even tiny missed bleeds may have devastating consequences, the bar for machine learning models to be accepted into practice is exceedingly high. In addition, algorithms must be able to generalize even after attaining strong performance, maintaining high accuracy on scans produced by different scanners at different institutions.

In recent years, computer vision advancements have led to algorithms that can detect (1–4) and segment intracranial hemorrhage (5). The detection capability of such models can help streamline the triage process in radiologic workflow while the segmentation capability can provide information about lesion localization and size, which are critical factors in downstream clinical management decisions. Segmentations can also be used for lesion quantification, which can answer important questions about brain injury outcomes. A recent study demonstrated that PatchFCN (6), a fully convolutional neural network trained on ~10^5^ images with pixel-level reference standard segmentations, achieved expert-level examination classification accuracy when tested on within-institution data. However, it is unclear whether such models can maintain expert-level accuracies on data from diverse sources. As variations in CT scanner hardware and technical parameters across institutions produce images with differing noise characteristics and image artifacts, models trained on data from one institution typically suffer performance losses when applied to data from other institutions. Therefore, improving a model’s generalization capability is critical for it to be widely deployed.

To address this issue, we propose the use of semi-supervised learning, a machine learning strategy that uses both labeled and unlabeled data at training time. Noisy student (7), a semi-supervised learning paradigm, demonstrated state-of-the-art performance on ImageNet, a widely used computer vision benchmark. Although semi-supervised learning has demonstrated promise on a variety of medical imaging applications (8,9), it has not yet been applied for the purpose of improving the generalizability of brain hemorrhage detection models (4,10) on out-of-distribution datasets. In this study, we applied the semi-supervised noisy student learning paradigm (7) to detect and segment intracranial hemorrhage on head CT images. We demonstrate that allowing a model to learn from a broader complement of both labeled and unlabeled data improves its generalizability and performance on out-of-institution datasets.

## Materials and Methods

All CT examinations were collected retrospectively in Digital Imaging and Communications in Medicine (DICOM) format. Based on U.S. regulation 45 CFR 46.116 (d) and the U.S. Food and Drug Administration, this study satisfied conditions for ethically acceptable waiver of patient consent and was approved by the institutional review board at the authors’ institution. The study protocol was approved by the University of California San Francisco Committee on Human Research and is Health Insurance Portability and Accountability Act compliant. All segmentations were performed using an in-house custom graphical user interface application written in Python created by our group.

### Datasets

The Atlantis-labeled dataset consists of 457 pixel-labeled clinical head CT scans performed from 2010 to 2017 with four 64–detector row CT scanners from a single vendor at a single institution. It contains the typical spectrum of pathoanatomic types of intracranial hemorrhage and image artifacts seen in clinical practice, with skull, scalp, and face removed for anonymity. In the Atlantis training dataset, 26.9% (123 of 457) of examinations are positive. Although this positivity rate is higher than that in the real world, it satisfies the need to include a sufficiently large sample of positive examinations and positive pixels. By hemorrhage subtype, 18.2% (83 of 457) of examinations contained subarachnoid hemorrhage, 14.0% (64 of 457) contained subdural hematoma, 13.3% (61 of 457) contained contusion, 5.0% (23 of 457) contained epidural hematoma, and 1.5% (seven of 457) contained intraventricular hematoma. Two board-certified neuroradiologists with 15 and 10 years of experience annotated all areas of hemorrhage at the pixel level. In an earlier study, data from all 457 CT examinations contained in the Atlantis dataset were also used to train PatchFCN (6). However, the goal of this prior study was to evaluate the classification accuracy of the model benchmarked against board-certified radiologists, while the present study seeks to use semi-supervised learning to improve model generalizability on out-of-distribution datasets.

The unlabeled training dataset Kaggle-25K was curated by the Radiological Society of North America (RSNA) and the American Society of Neuroradiology and consists of an external corpus of more than 25 000 head CT examinations from the Kaggle RSNA Intracranial Hemorrhage Detection competition (11). Kaggle-25K contains image-level labels but was treated as an unlabeled dataset for the purpose of semi-supervised learning. Data annotators can be found in Stein et al (11).

For model development, we collected a validation dataset for hyperparameter tuning consisting of 93 head CT scans performed at hospitals not represented in either the labeled or unlabeled datasets. Of the 93 head scans, 10.8% (10 of 93) were positive. All scans were read by E.L.Y., a board-certified neuroradiologist with 14 years of experience.

For testing, we used the CQ500 dataset (2), curated by Qure.ai and the Center for Advanced Research in Imaging, Neurosciences and Genomics in New Delhi, India, and licensed under a Creative Commons Attribution-NonCommercial-ShareAlike 4.0 International license and end-user license agreement. This public dataset includes 491 examinations with reads from three radiologists with 8, 12, and 20 years of experience. We used a majority vote of the three radiologists to produce reference standard examination-level labels. The CQ500 test set contained 41.8% (205 of 491) positive examinations. The full spectrum of acute intracranial hemorrhage was included in CQ500, with 27.3% (134 of 491) of examinations containing intraparenchymal hemorrhage, 12.2% (60 of 491) containing subarachnoid hemorrhage, 10.8% (53 of 491) containing subdural hematoma, 5.7% (28 of 491) containing intraventricular hemorrhage, and 2.6% (13 of 491) containing epidural hematoma. Ten examinations that were chest or abdominal CT examinations and were missing sections or consisted of 0.6-mm sections not designed for human interpretation were excluded. To evaluate pixel accuracy, we randomly selected and pixel-labeled a 23-examination subset (*k* = 529 images) that reflected the positive examination rate of the overall dataset. We annotated a subset because pixel labeling the full dataset was prohibitively expensive and time-consuming. Results on CQ500 have previously been published by the dataset curators (2), but we used CQ500 as an out-of-distribution test set for the purpose of evaluating our model’s generalization capabilities. The data can be accessed at *http://headctstudy.qure.ai/dataset*.

### Semi-supervised Algorithm Development

**Figure 1:**
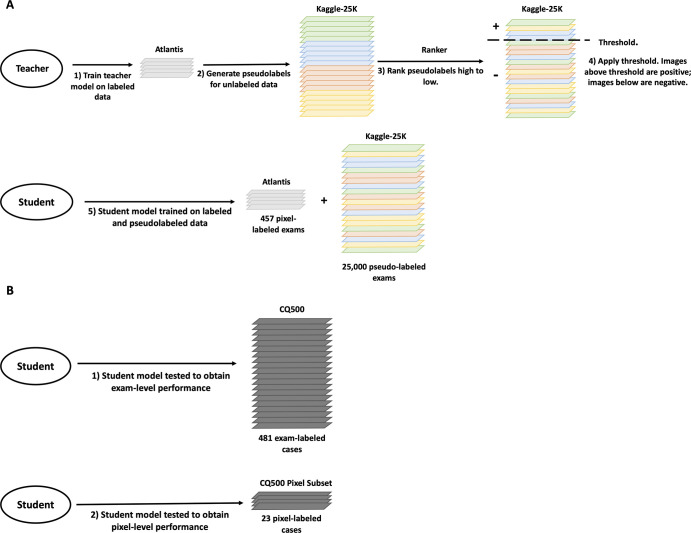
Schematic of the semi-supervised noisy student approach. Each color signifies data from a different institution. **(A)** Schematic shows the workflow at training time, which is explained in further detail in the Semi-supervised Algorithm Development section. **(B)** Schematic shows the workflow at test time as the student model is evaluated on both the CQ500 overall dataset and pixel-label subset to evaluate examination-level and pixel-level performances.

***Overview.—*** We applied the noisy student (7) learning paradigm. The training workflow (Fig 1A) is as follows: *(a)* train a teacher model on a small pixel-labeled dataset; *(b)* the trained teacher model generates pixel-level and image-level pseudo labels, or predictions, on a large unlabeled dataset; *(c)* rank pseudo-labeled images from high to low based on probability of hemorrhage; *(d)* apply a threshold, setting all images above the threshold to positive and all remaining images to negative; and *(e)* train a student model on the combination of the small pixel-labeled dataset and the larger pseudo-labeled dataset.

This trained student model was then tested on the overall CQ500 dataset and the pixel-labeled CQ500 subset to evaluate both examination-level and pixel-level performances, respectively (Fig 1B). The teacher model was trained from scratch, while the student model was initialized based on the weights of the teacher model as per the noisy student paradigm.

***Data augmentation.—*** One important element in the noisy student approach (7) is the use of data augmentation to help achieve optimal performance. We explored three augmentation strategies: adjustments of image contrast, head size, and head aspect ratio. To employ contrast adjustment, each image in the training set underwent power law transformation, logarithmic correction, or no change (12,13), with a random one-third selection probability for each. To modify head size and aspect ratio, the lengths and widths of each head were independently adjusted. Final augmentation parameters were selected based on the values that yielded the best performance on the validation dataset.

**Figure 2:**
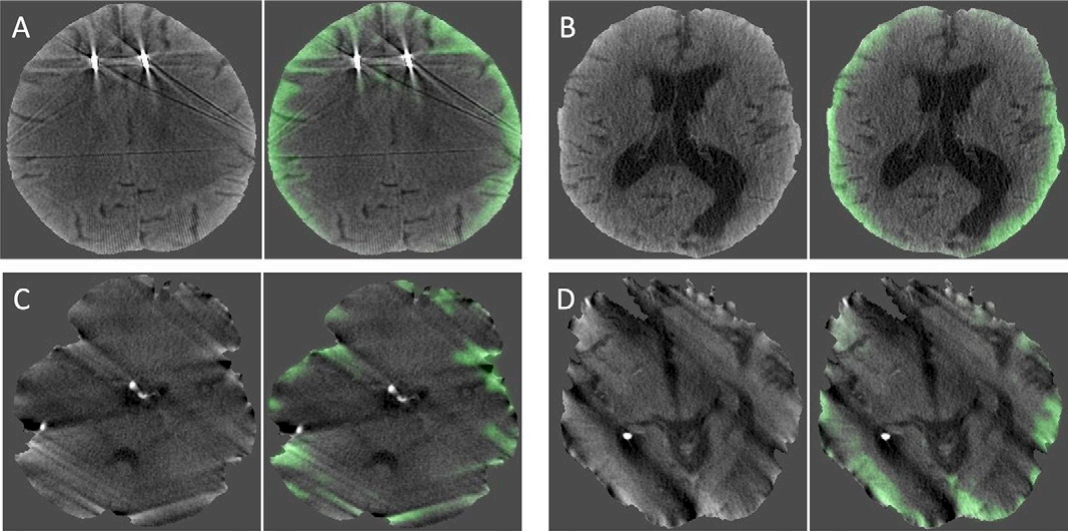
**(A–D)** Images show visualization of false-positive predictions, which are frequently made without the ranker. Green indicates the model predictions. These axial CT images obtained without contrast material administration are from the validation set.

***Ranker.—*** We incorporated a ranker to reduce false-positive errors, a common error type (Fig 2). First, the teacher model generates hemorrhage prediction probabilities for each unlabeled image. The images from the unlabeled dataset are ordered from high to low based on the hemorrhage probability predictions. A threshold is applied based on the teacher’s predictions, with images above the threshold considered positive and images below the threshold considered negative. To select the ranker threshold for semi-supervised model training, a radiologist first inspected a small set of images from the unlabeled dataset at various percentile thresholds *C* ∈ {5, 10, 15, 20, 25, 30}. At the ideal threshold, images with probabilities above the threshold contain true positives without false positives. After inspection, we selected a threshold of *C* = 10. Therefore, only the 10% of images with the greatest probability of hemorrhage were considered positive. For positive images, we set pixels with confidences of *K* greater than 0.7 to positive and *K* less than 0.3 to negative. Values in between are ignored and generate no loss during training. All other images were considered negative, with all pixel predictions set to 0.

***Implementation details.—*** The semi-supervised model was developed on PatchFCN (6), a fully convolutional neural network with a Dilated ResNet-38 backbone architecture and was initialized randomly. The PatchFCN model employs two branches, a classification branch and a segmentation branch which leverage image- and pixel-level losses, respectively. At training time, the model takes a patch of an image as input. The classification branch is supervised by patch-level binary class labels, while the segmentation branch is supervised by pixel labels. The patch-level class labels are derived from the pixel labels by checking whether the patch contains any positive pixel. Examination-level labels are not used in segmentation model training and are used for evaluation only. At inference time, PatchFCN takes a patch of an image as an input and outputs a binary class label (hemorrhage vs no hemorrhage) and segmentation pixel probability scores. The student model trained for 600 epochs with a step number of 240, batch size of 16, and a crop size of 240. We used a mixing ratio of 0.6:0.4 of Atlantis to Kaggle-25K for each minibatch during training. More details about PatchFCN may be found here: *https://doi.org/10.1073/pnas.1908021116*. The model was developed in PyTorch based on the publicly available code, *https://github.com/fyu/drn*. Experiments were performed on a single NVIDIA Tesla V100 GPU (NVIDIA).

### Models

The baseline model consists of the PatchFCN model trained on the Atlantis pixel-labeled dataset only. The semi-supervised model consists of the PatchFCN model trained on both the Atlantis and Kaggle-25K dataset using the noisy student paradigm. Kaggle-25K was treated as an unlabeled dataset, and pixel pseudo labels for this data were generated through the noisy student process.

Although the primary point of comparison is between the baseline and semi-supervised models, we also included the Oracle models as additional performance benchmarks. Oracle models are those that are trained with additional labels beyond what would normally be available in a semi-supervised learning paradigm. The image section Oracle model, like the semi-supervised model, was trained on Atlantis and Kaggle-25K. However, it employs additional reference standard image labels that were curated and publicly available for Kaggle-25K, as this dataset did not come with pixel-level labels. At training time, pixel loss is activated on Atlantis only. On Kaggle-25K, the whole image is used as a patch, and the reference standard image label is equivalent to the patch label. The pixel Oracle model goes one step further. On Kaggle-25K, it is trained with both the reference standard image labels and the semi-supervised model’s pixel pseudo labels. Pixel loss is activated on both Atlantis and Kaggle-25K, and reference labels are used to suppress false-positive predictions. If the reference standard image label is negative, all pixel pseudo labels for that image are set to 0. If the reference standard image label is positive, the teacher model’s pseudo-label predictions are used. However, in a real-world clinical setting, the vast majority of data are unlabeled and do not have image or pixel labels. Therefore, while the pixel Oracle model represents the upper bound of performance and provides insight to assess the best achievable performance given the available data and labels, the primary comparison is between the baseline and semi-supervised models.

The Qure.ai model was developed by the curators of CQ500. We used this as an additional performance benchmark.

### Statistical Analysis

The examination area under the receiver operating characteristic curve (AUC) reflects examination-level classification accuracy. To compare examination-level performance between the baseline and semi-supervised models, we collected 1000 sets of 481 CT examinations each by conducting random sampling with replacement on the overall CQ500 dataset. We obtained ΔAUC, the estimated differences in examination AUC between the baseline and semi-supervised models, on all 1000 bootstrapped sets of 481 CT examinations and confirmed that the ΔAUC are normally distributed at a confidence level of *P* less than .05 using a standard Kolmogorov-Smirnov test for normality. We then calculated the test statistic (*Z* = ΔAUC/σ, where σ is the empirical SD of ΔAUC on the 1000 bootstrapped sets of 481 CT examinations) and calculated a two-sided *P* value as twice the probability that a standard normal random variable is less than |*Z*|. We also reported the 95% CI of the ΔAUC between models as the 2.5th and 97.5th percentile values of ΔAUC.

To compare pixel-level performance between the baseline and semi-supervised models, we collected 1000 sets of 529 images by conducting random sampling with replacement on the images comprising the pixel-labeled CQ500 test set. We obtained the estimated differences in the Dice similarity coefficient (ΔDSC) between the baseline and semi-supervised models on all 1000 bootstrapped sets of 529 images and confirmed that the ΔDSC are normally distributed at a confidence level of *P* less than .05 using a standard Kolmogorov-Smirnov test for normality. We then calculated test statistic (*Z* = ΔDSC/σ, where σ is the empirical SD of the DSC differences on the 1000 bootstrapped sets of 529 images) and calculated a two-sided *P* value as twice the probability that a standard normal random variable is less than |*Z*|. We also reported the 95% CI of ΔDSC between models as the 2.5th and 97.5th percentile values of ΔDSC. We obtained the difference in pixel average precision (ΔAP) between baseline and semi-supervised models in similar fashion and reported the 95% CI of ΔAP between models as the 2.5th and 97.5th percentile values of ΔAP.

### Intrarater Reliability

The DSC and pixel AP (14) segmentation metrics provide a measure of the localization capability of the system. To evaluate the segmentation results more rigorously and verify the superiority of the semi-supervised model compared with the baseline, we performed test-retest segmentations using a single labeler. The same board-certified neuroradiologist segmented the same 23-examination test set (529 images) a total of three times. Each segmentation was performed without review of prior segmentations with a washout period of at least 7 days between examinations and random reordering of examinations.

To quantitatively determine whether intrarater variability affects our results, we calculated the pixel AP and DSC for the baseline model on 50 bootstrapped samples on each of the three neuroradiologist segmentations. This process was repeated for the semi-supervised model.

## Results

### Patient Demographics

The semi-supervised model was trained on both the Atlantis and Kaggle-25K datasets. For the Atlantis dataset, the mean age of the patients was 53.4 years ± 17.1 [SD], and 36.3% (166 of 457) of the patients were female. Patient demographics for Kaggle-25K were not released. Main results were presented on CQ500, which was collected in two batches, B1 (43.9% [94 of 214] female patients; mean age, 43.4 years) and B2 (30.3% [84 of 277] female patients; mean age, 51.7 years).

### Model Performance Comparison on CQ500

The semi-supervised model achieved a significant performance gain over the baseline model on the CQ500 test set across AUC, DSC, and pixel AP metrics, demonstrating both superior localization and examination-level decision capabilities over the baseline (Table 1). For the detection metric, the semi-supervised model demonstrates an examination AUC of 0.939 (95% CI: 0.938, 0.940), performing significantly better than the baseline examination model which had an AUC of 0.907 (95% CI: 0.906, 0.908; *P* < .001) (Fig S1). The Qure.ai model (2), which yields state-of-the-art performance on CQ500, achieves an examination AUC of 0.941. In addition, using an operating point on the AUC curve at which the sensitivity is 0.920 on the CQ500 test set, the baseline and semi-supervised models showed a specificity of 0.630 and 0.804, respectively. The semi-supervised model also surpassed performance of the image section Oracle model (0.894; *P* < .001) and nearly matched that of the pixel Oracle (0.943). Finally, the semi-supervised model showed improvements over the baseline model for classification of hemorrhage subtypes (Table S1).

**Table 1:**
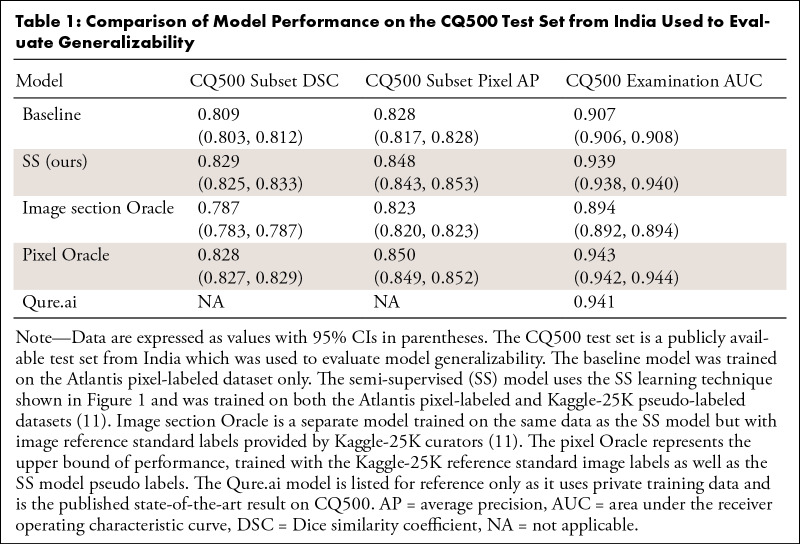
Comparison of Model Performance on the CQ500 Test Set from India Used to Evaluate Generalizability

The DSC of the semi-supervised model was 0.829 (95% CI: 0.825, 0.833), a significant improvement over the baseline DSC of 0.809 (95% CI: 0.803, 0.812; *P* < .001). The semi-supervised model demonstrated a pixel AP of 0.848 (95% CI: 0.843, 0.853), a significant improvement over the baseline pixel AP (0.828; 95% CI: 0.817, 0.828) as well the image section Oracle AP (0.823; 95% CI: 0.843, 0.853) and a near match to the pixel Oracle AP (0.850).

**Figure 3:**
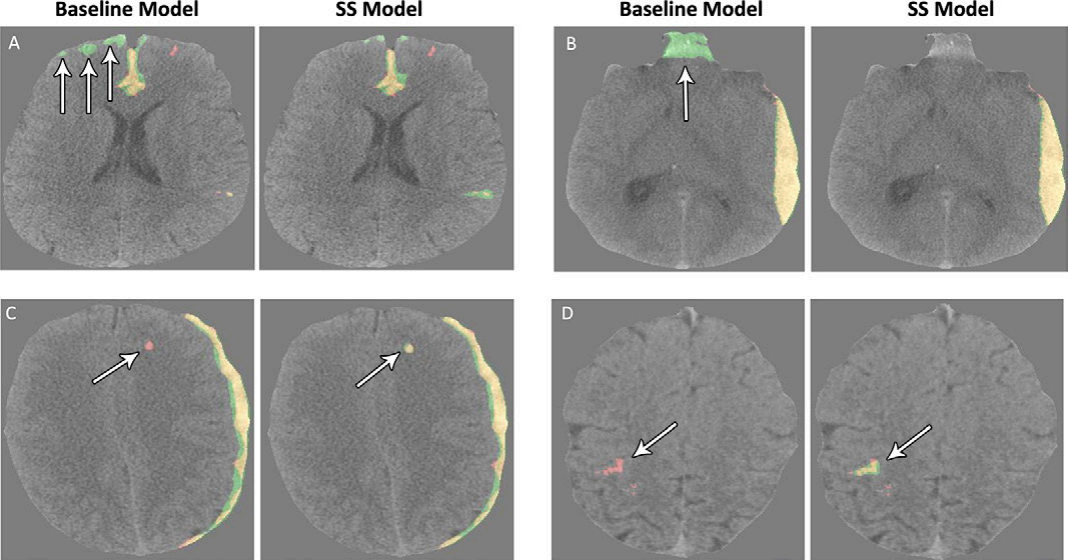
**(A–D)** Images show visualization of model predictions on the validation set using the baseline and semi-supervised (SS) models. Red is the reference standard label, green is the model’s positive prediction, and yellow is the overlap. These are axial CT images obtained without contrast material administration.

Corresponding to the results on the metrics above, the semi-supervised model demonstrated increased ability to detect subtle bleeds and reject false positives. Figure 3 shows baseline and semi-supervised model prediction visualizations on the validation set. The semi-supervised model was more robust against false positives, recognizing many of the baseline model’s false-positive predictions as true negatives (Fig 3A, 3B). It was also able to accurately detect bleeds that the baseline model missed (Fig 3C, 3D).

### Data Augmentation Parameter Optimization

To identify the best data augmentation strategy, we investigated model performance using different contrast parameters (Table 2). Because Kaggle-25K pseudo labels are generated at the pixel level, we optimized for pixel AP. The parameters were fine-tuned on the baseline and evaluated on the validation set. The baseline pixel AP was 0.651. We then implemented power law and logarithmic transformations individually which yielded pixel AP values of 0.653 and 0.649, respectively. Finally, we studied whether both strategies produced an additive effect by combining power law, logarithmic transformation, and no augmentation with equal sampling ratios. Out of all contrast augmentation schemes, the combination strategy yielded the best pixel-level performance (0.681) on the validation set. Therefore, we selected this combined data augmentation strategy as the one to use for the semi-supervised model.

**Table 2:**
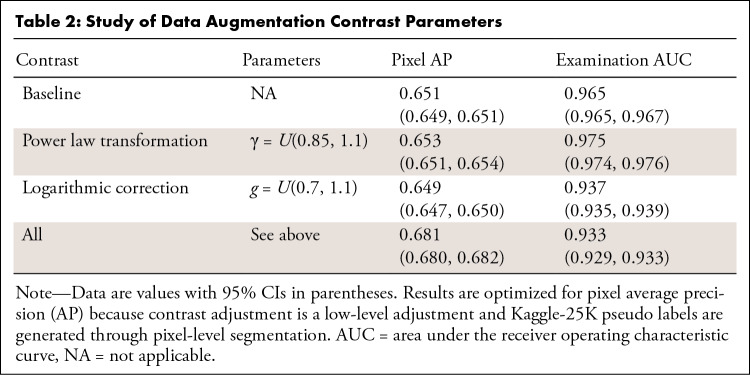
Study of Data Augmentation Contrast Parameters

### Ranker Ablation Study

Table 3 presents the results of our control study investigating the importance of a ranker for the semi-supervised model.

**Table 3:**
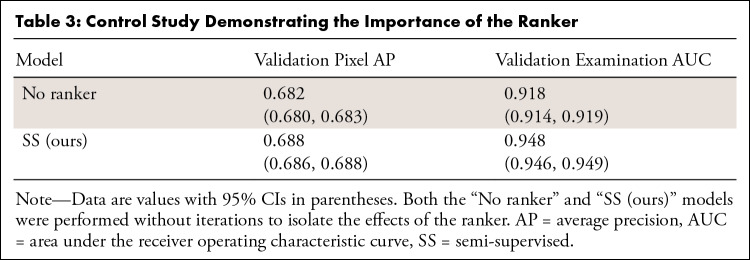
Control Study Demonstrating the Importance of the Ranker

If the ranker is removed, the examination AUC drops from 0.948 to 0.918, as the model often generates pseudo labels containing false-positive predictions (Fig 3) that are suppressed by the ranker. However, without the ranker, the student would train on and reinforce its own false-positive predictions.

### Test-Retest Reliability

We found a statistically significant superiority of the semi-supervised model over the baseline model on each of the three neuroradiologist segmentations on both pixel AP and DSC (Table 4). Paired *t* tests comparing the baseline and semi-supervised model on all three segmentations for pixel AP and DSC produced *P* values less than .001.

**Table 4:**
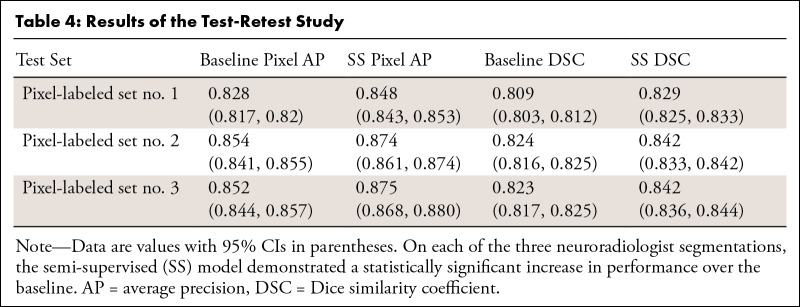
Results of the Test-Retest Study

## Discussion

One major barrier to the widespread clinical deployment of machine learning models is the need for improved generalization. Models must maintain high levels of performance accuracy on data from diverse sources encompassing heterogeneous populations and differing technical parameters. To address this, we employed a semi-supervised learning paradigm to detect and segment intracranial hemorrhage on head CT images using a broad complement of both labeled and unlabeled data to improve model generalization capability. We demonstrated the potential for semi-supervised learning to significantly improve both granular and holistic localization capabilities. The examination AUC of the semi-supervised model (0.939) was significantly stronger than that of the baseline model (0.907). The DSC of the semi-supervised model was 0.829, which demonstrated a significant improvement over the baseline DSC of 0.809. The semi-supervised model demonstrated a pixel AP of 0.848, a significant improvement over the baseline pixel AP of 0.828. These results are qualitatively supported by the baseline and semi-supervised model output visualizations in which the semi-supervised model demonstrated both increased sensitivity and specificity of predictions. The semi-supervised model (examination AUC, 0.939) was also able to achieve performance similar to that of Qure.ai (examination AUC, 0.941) on the CQ500 test set despite using significantly more out-of-distribution data and 640-fold fewer labeled examinations.

The image section Oracle and pixel Oracle are provided as a means of comparison. The semi-supervised model likely outperforms the image section Oracle because the pixel pseudo labels on Kaggle-25K provide superior localization information. Although the pixel Oracle performance minimally surpasses that of the semi-supervised model, it requires the cost of 285 000 extra image-level labels provided by experts. The semi-supervised model achieved very similar performance without the need for the time and monetary cost required to obtain these image-level labels.

We also demonstrated how important both data augmentation and the ranker are to maintaining strong performance. Data augmentation is helpful during training time because it exposes the model to greater data distribution, thereby enhancing its generalization capability. CT scans may vary in patient head aspect ratios and head sizes, so exposure to a wider data distribution would allow the model to learn from a broader and therefore challenging set of examples. The introduction of the ranker is also important. The majority of head CT scans are administered as cautionary screening tools and are therefore negative. The ranker operationalizes this prior clinical knowledge to minimize false-positive errors, ultimately improving performance.

Because our goal was to evaluate generalizability, we selected the CQ500 test set which was curated on a different continent. This is a challenging setting for the model as the data included scans performed on previously unfamiliar populations with exposure to additional artifacts and technical characteristics that had not previously been encountered.

To our knowledge, semi-supervised learning has not been applied for generalizability of hemorrhage detection in deep learning models. Salehinejad et al (15) leveraged the RSNA Intracranial Hemorrhage CT dataset to explore the generalizability of their intracranial hemorrhage machine learning model. Remedios et al (16) applied cyclic weight transfer to improve generalization for head CT hemorrhage segmentation. However, both approaches require all training data to be labeled and are unable to support the aggregation of labeled and unlabeled data as seen in semi-supervised learning. Additionally, neither model can be compared directly with ours due to differences regarding the choice of training and evaluation sets. Outside of these works, generalizability remains largely unexplored for this application. Wang et al (17) have applied semi-supervised learning for intracranial hemorrhage segmentation, but they do not leverage this strategy for improved generalization. Many other works also exist to develop high-performing models for intracranial hemorrhage (2,4,6), but they do not tackle the issue of generalizability.

This study, however, still contains some limitations. In semi-supervised learning paradigms, the size of the unlabeled corpus is significantly larger than that of the labeled corpus. As a result, there is a greater computational requirement to process and train the large volumes of unlabeled data. As the amount of data increases, the required computational power and training time also increases. In addition, both labeled and unlabeled datasets typically grow with time in real-world clinical settings. The current workflow would require the algorithm to be completely retrained from scratch with every increase in dataset size, which is a time-consuming process. To optimize this system for application, more research will be required to develop a semi-supervised learning scheme that requires minimal adaptation training on growing datasets.

In conclusion, we demonstrated that a semi-supervised learning paradigm could help achieve stronger generalization for intracranial hemorrhage classification and segmentation at head CT. We demonstrated the potential for algorithms to improve performance by aggregating both labeled and unlabeled data. While annotated data are valuable toward achieving strong performance, they also require substantial time and energy to obtain. In contrast, unlabeled data can be found in abundance in clinical settings and are essentially “free” in its lack of requirement for any annotation time. Once an algorithm is seeded with a small high-quality labeled dataset, a semi-supervised learning paradigm can raise the performance level simply by leveraging the vast unlabeled data in the real-world setting. The addition of unlabeled data through a semi-supervised approach can thus serve as a useful tool to improve generalizability and augment performance with minimal additional cost.
